# Serial Cerebral Metabolic Changes in Patients With Ischemic Stroke Treated With Autologous Bone Marrow Derived Mononuclear Cells

**DOI:** 10.3389/fneur.2019.00141

**Published:** 2019-02-25

**Authors:** Muhammad E. Haque, Refaat E. Gabr, Sarah D. George, Seth B. Boren, Farhaan S. Vahidy, Xu Zhang, Octavio D. Arevalo, Susan Alderman, Ponnada A. Narayana, Khader M. Hasan, Elliott R. Friedman, Clark W. Sitton, Sean I. Savitz

**Affiliations:** ^1^Institute for Stroke and Cerebrovascular Diseases, McGovern Medical School, The University of Texas Health Science Center at Houston, Houston, TX, United States; ^2^Diagnostic and Interventional Imaging, McGovern Medical School, The University of Texas Health Science Center at Houston, Houston, TX, United States; ^3^Biostatistics, Epidemiology, Research Design Component, Center for Clinical and Translational Sciences, McGovern Medical School, The University of Texas Health Science Center at Houston, Houston, TX, United States

**Keywords:** autologous mononuclear cells transplantation, cells therapy, ischemic stroke, cerebral metabolites, magnetic resonance spectroscopy

## Abstract

**Purpose:** Cell-based therapy offers new opportunities for the development of novel treatments to promote tissue repair, functional restoration, and cerebral metabolic balance. N-acetylasperate (NAA), Choline (Cho), and Creatine (Cr) are three major metabolites seen on proton magnetic resonance spectroscopy (MRS) that play a vital role in balancing the biochemical processes and are suggested as markers of recovery. In this preliminary study, we serially monitored changes in these metabolites in ischemic stroke patients who were treated with autologous bone marrow-derived mononuclear cells (MNCs) using non-invasive MRS.

**Materials and Methods:** A sub-group of nine patients (3 male, 6 female) participated in a serial MRS study, as part of a clinical trial on autologous bone marrow cell therapy in acute ischemic stroke. Seven to ten million mononuclear cells were isolated from the patient's bone marrow and administered intravenously within 72 h of onset of injury. MRS data were obtained at 1, 3, and 6 months using a whole-body 3.0T MRI. Single voxel point-resolved spectroscopy (PRESS) was obtained within the lesion and contralesional gray matter. Spectral analysis was done using TARQUIN software and absolute concentration of NAA, Cho, and Cr was determined. National Institute of Health Stroke Scale (NIHSS) was serially recoreded. Two-way analysis of variance was performed and *p* < 0.05 considered statistically significant.

**Results:** All metabolites showed statistically significant or clear trends toward lower ipsilesional concentrations compared to the contralesional side at all time points. Statistically significant reductions were found in ipsilesional NAA at 1M and 3M, Cho at 6M, and Cr at 1M and 6M (*p* < 0.03), compared to the contralesional side. Temporally, ipsilesional NAA increased between 3M and 6M (*p* < 0.01). On the other hand, ipsilesional Cho showed continued decline till 6M (*p* < 0.01). Ipsilesional Cr was stable over time. Contralesional metabolites were relatively stable over time, with only Cr showing a reduction 3M (*p* < 0.02). There was a significant (*p* < 0.03) correlation between ipsilesional NAA and NIHSS at 3M follow-up.

**Conclusion:** Serial changes in metabolites suggest that MRS can be applied to monitor therapeutic changes. Post-treatment increasing trends of NAA concentration and significant correlation with NIHSS support a potential therapeutic effect.

## Introduction

The only therapeutic options for patients with ischemic stroke are approaches that promote recanalization and reperfusion to restore blood flow to the brain. Currently, there are no effective post stroke interventions that can promote repair of damaged tissues and restore brain function. Cell based therapies offer great potential to promote possible repair of damaged tissue, prevent atrophy, and help restore brain function. Extensive animal data have shown the safety and feasibility of the bone marrow-derived mononuclear cells (MNCs) as an attractive therapeutic option for stroke (1–4). Significant physical and cognitive improvement was shown to occurs in animals treated with cell therapies by providing a protective mechanism to attenuate progressive tissue damage (5–8). Clinical trials have shown the feasibility and safety of both autologous and allogenic cell administration in stroke patients (9–13). An important advancement in this field would be to develop post-stroke therapeutic biomarkers.

Number of post-stroke magnetic resonance spectroscopy (MRS) studies have shown elevated lactate (Lac), decreased N-acetylaspartate (NAA) and Creatine (Cr), and variable changes in choline (Cho) concentration in the ipsilesional hemisphere (14–18). Restoration of these cerebral metabolites concentration could facilitate in the evaluation of recovery (19). NAA is synthesized in neurons and is crucial for cerebral lipid synthesis and energy production (20, 21). The cerebral Cr is associated with energy recycling via converting adenosine diphosphate (ADP) to ATP (22). Choline is involved in memory function and muscle control (23). These metabolites have been extensively used as surrogate markers of neuronal health (NAA), membrane integrity (Cho), cellular energy (Cr) and oxidative stress (Lac) (14, 16, 24–28).

The application of MRS to characterize neural progenitor cells (NPCs) *in-vivo*, which can differentiate into neurons, astrocytes, and oligodendrocytes, has been reported (29). Furthermore, two separate studies presented MRS as a tool for monitoring NPCs and human umbilical mesenchymal stem cells differentiation (30, 31). Number of *in-vitro* studies showed the ability of MRS to identify cell types based on their metabolic profile in the cell culture (31). Previously, Brazzini reported an increase in NAA concentration in both left and right basal ganglia in patients with Parkinson treated with bone marrow-derived autologous MNCs transplantation (32). Metabolic improvement was also documented in amyotrophic lateral sclerosis patients treated with stem cells (33).

In this prospective study, we serially monitored NAA, Cho, and Cr concentrations with MRS as possible therapeutic biomarkers following intravenous infusion of autologous MNCs in patients with acute ischemic stroke. Our findings in this preliminary study encourage pursuing a larger study that can be correlated with therapeutic efficacy to predict post-stroke recovery.

## Materials and Methods

### Human Protection

This study was conducted under Federal Investigational New Drug Application BB IND 13775 and was approved by the University of Texas Health Sciences Center at Houston Committee for the Protection of Human Subjects and by the Memorial Hermann Hospital Office of Research.

### Patient Enrollment and Serial MRI

A sub-group of nine patients (3 males, 6 females) participated in this serial MRS study, which was an add-on scan to clinical trial testing autologous bone marrow mononuclear cells in patients with ischemic stroke. Written informed consent was obtained after a thorough discussion with the patient family prior to enrollment. The inclusion and exclusion criteria are described elsewhere (10). Post MNCs follow-up imaging was done at one (1M), three (3M), and six (6M) months of onset.

### Intervention

#### Bone Marrow Cells Harvesting

The procedural details about mononuclear cells isolation and intravenous infusion are described elsewhere (10). Briefly, a total of 2 ml/kg bone marrow was harvested aseptically from the posterior iliac bone. All patients were placed in the prone position and a 11-gauge bone marrow needle was placed into the posterior iliac crest, and about 5 to 7 cc was aspirated with a 20-cc syringe and the procedure repeated. IV normal saline was administered if there were changes in the patient's hemodynamics, and all the patients were monitored for oxygen saturation, blood pressure, heart and respiratory rate before, during and after the procedure.

#### MNCs Isolation and Infusion

Bone marrow was transported in an anticoagulated blood collection bag for isolation and enrichment of the MNCs. The bone marrow was filtered (170-ml blood filter) to remove spicules. The MNCs were enriched from the bone marrow using Ficoll-Paque (GE Healthcare, Milwaukee, WI) and Plus density gradient separation using the density gradient procedure on the Sepax device (Biosafe SA, Geneva, Switzerland). The MNCs were then washed twice with 5% human serum albumin in normal saline and adjusted to the appropriate concentration for administration (final concentration of 1.25%). A maximum of 10 million cells/kg in normal saline was administered into the antecubital vein over 30 min. Cells Transplantation was done within 72 h from onset of the stroke.

#### Clinical Assessments

National Institutes of Health Stroke Scale (NIHSS) (34), modified Rankin scale (mRS), and Barthal Index (BI) were recorded at baseline and NIHSS at each follow-up visit.

#### Imaging Acquisition Protocols

All imaging experiments were performed on a whole body 3.0 T Philips system (Philips Healthcare, Best, The Netherlands) using 8-channel head coil. Structural MRI was obtained using 3D T1-weighted volumes (TE/TR = 3.66 ms/8.2 ms, acquisition matrix = 256 × 256 × 170, FOV = 256 × 256 mm^2^) and T2-weighted (TE/TR/ = 80 ms/2.5 s, acquisition matrix = 256 × 256 × 170, and FOV = 256 × 256 mm^2^) images. Anatomical localizations of the lesions were performed using 2D fluid-attenuated inversion recovery (FLAIR, TE/TI/TR = 95 ms/2.6 s/11 sec, acquisition matrix = 256 × 256, FOV = 256 × 256 mm^2^) images. Single voxel (SV) proton MRS was obtained with point-resolved spectroscopy (PRESS, voxel size 20 × 20 × 20 mm^3^ TE/TR = 35/2,000 ms, sampling frequency = 2,000 Hz, data points = 1,024, number of scans = 128) on both the ipsilesional and contralesional regions. Volumes of interest (VOIs) were carefully placed within the infarct and contralateral normal appearing gray matter tissue using FLAIR images. Standard 3-pulse chemical shift selective scheme was used for water suppression with automated shimming. Unsuppressed water spectra were also obtained as an internal reference for metabolite quantification. Saturation bands were placed around the VOI to minimize lipid contamination.

#### Spectral Analysis and Metabolite Quantification

All the spectral analysis were performed using publicly available Totally Automated Robust Quantitation in NMR (TARQUIN) software (35), available at https://www.nitrc.org/projects/tarquin/. Absolute concentration was calculated in milli-mole (mM) units by scaling the fitted signal amplitude by the amplitude of the unsuppressed water signal. A detailed description of the measurements are described elsewhere (36, 37). Metabolite peaks at 2.01, 3.03, 3.19 ppm were referenced to NAA, Cr, and Cho, respectively, with respect to unsuppressed water signal at 4.7 ppm. Typically metabolite concentrations are normalized to creatine or choline concentration assuming they were constant; here, we reported absolute concentrations because they were not constant.

#### Lesion Volume Measurements

A semi-automated seed growing algorithm within Analyze 12.0 (Analyze Direct Inc., KS, USA) was used to delineate lesion volume on T2-weighted images by a single rater. The rater selected two seed points within the hyper and hypointense regions within the lesions and a region-growing algorithm automatically expanded the seed points within the 3D space of the image. Manual editing of the lesion volume was done when necessary.

## Statistical Analysis

Cerebral metabolites concentration was analyzed by the mixed model (38) The fixed effects in the model included brain hemisphere (ipsilesional and contralesional), polychotomous time (1, 3, and 6 month), and interaction between hemisphere and polychotomous time. The random effects in the model included patient and interaction between patient and hemisphere. These random effects led to a nested covariance matrix accounting for correlation of measurements due to the same patient and a different level of correlation for measurements due to the same hemisphere of brain. Correlation between cerebral metabolites concentrations and NIHSS scores was depicted in scatter plot, together with Pearson correlation coefficient. Two-sided *p*-values were reported and *p*-values less than 0.05 were considered as significant. All statistical analyses were performed using the SAS software (version 9.4, the SAS Institute, Cary, NC).

## Results

After cellular intervention, six female and three male patients with average age of 56.5 ± 17.6 years (range 31–78 years) underwent serial metabolite measurements. Patient demographics and lesion laterality, size, location, and severity are summarized in Table 1. Patient P01 data were removed from spectroscopic measurement because of noisy spectra at two time points. The lesion size was 13.9–74.4 cc at 1M, and NIHSS was 5–34 at the onset. There was a significant (*p* < 0.03) decrease in lesion volume between 1 and 6 months.

**Table 1:** Patient demographics, clinical score, and lesion volume.

**PID**	**Laterality**	**Lesion location**	**NIHSS**				**Lesion volume (cm**^****3****^**)**		
			**Baseline**	**01M**	**03M**	**06M**	**01M**	**03M**	**06M**
P01	L	Insula, frontal lobe	14	3	3	1	74.4	67.6	68.6
P02	R	Insula, IFG	9	6	3	2	50.2	26.4	25.1
P03	L	Insula, IFG	10	1	1	1	23.6	14.1	25.13
P04	R	Putamen, GP, CR	15	7	5	2	33.1	15.1	13.8
P05	R	Putamen, GP, CR	34	7	5	4	22.2	8.0	7.12
P06	R	Putamen, insula, FG	17	7	9	11	27.5	26.4	28.4
P07	R	Putamen	8	6	5	5	17.9	7.54	6.54
P08	R	TL, OL	16	9	8	7	13.9	12.9	11.5
P09	L	ITG, STG, FG, insula	8	5	5	6	6.08	6.8	6.03

*IFG, Inferior Frontal Gyrus; GP, Globus Pallidus; CR, Corona Radiata; FG, Frontal Gyrus; TL, Temporal Lobe; OL, Occipital Lobe; ITG, Inferior Temporal Gyrus; STG, Superior Temporal Gyrus; NIHSS, National Institute of Health Stroke Scale*.

**Figure 1:**
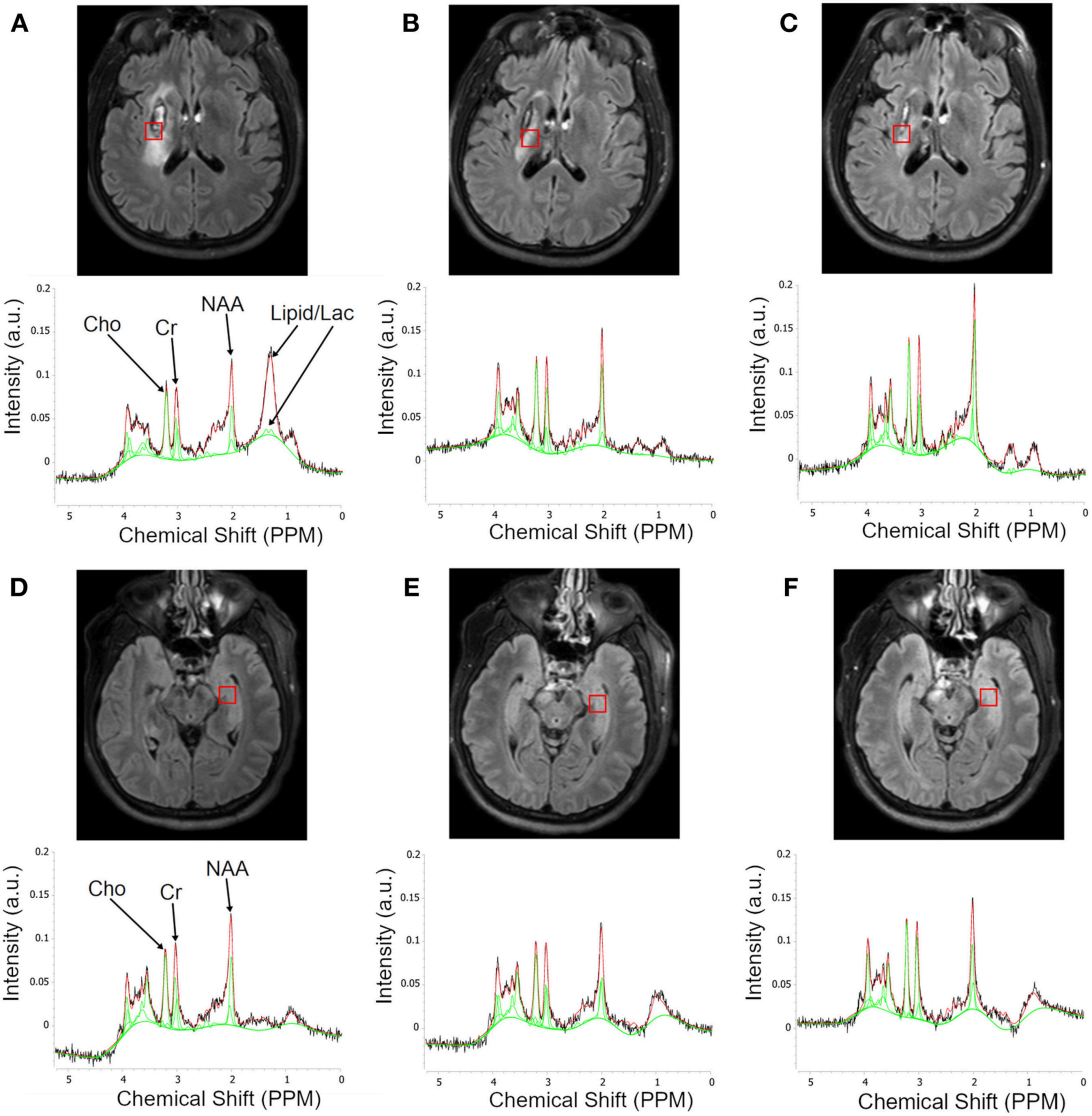
Serial Flair MR images illustrating typical MRS voxel placement in the lesion and contralateral tissues and corresponding MR spectra. The first row of images **(A–C)** is a single slice showing voxel placement in the lesion at 1, 3, and 6 months, respectively. The second row shows the corresponding spectra within the lesion. Each plot shows the raw spectrum (black), library spectra of matching metabolites (red), and fitted function (green). Third row **(D–F)** shows voxel placement in the contralesional gray matter with corresponding spectra in the fourth row. Note the questionable peak at 1.33 ppm at 1 month that disappears at the later time point.

Figure 1 shows a representative MRI of a patient illustrating typical MRS voxel placement in the lesion and contralateral tissue at the three follow-up scans, with their corresponding spectra below it. The contralateral voxel was placed on the normal appearing gray matter regions with some expected partial volume. Spectra were scaled and normalized to the unsuppressed water signal. As shown, the signal amplitude of NAA on the lesion spectra increased at 6 months as compared to 1 month, with minimal change in the contralateral NAA.

**Figure 2:**
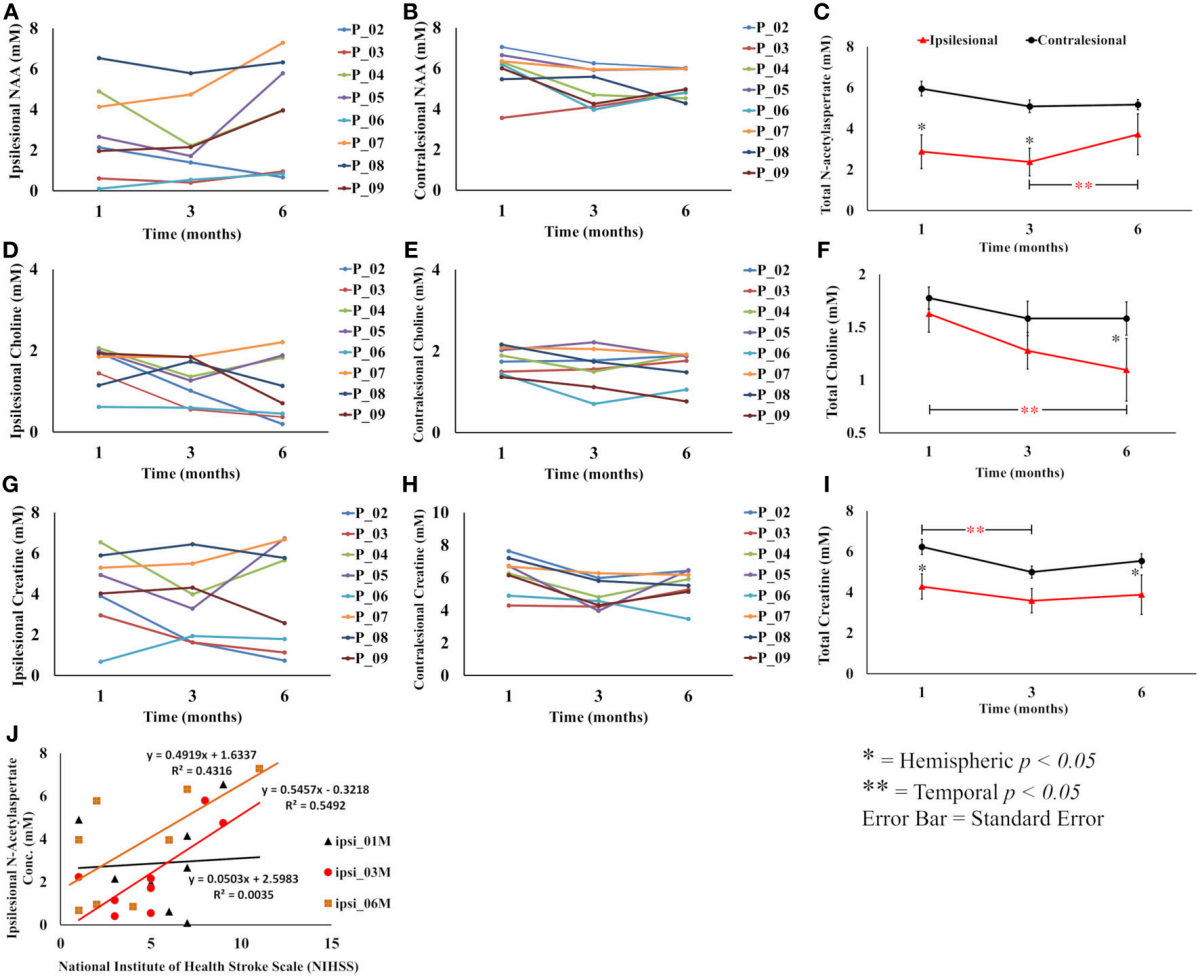
Overall summary of post treatment serial change in metabolite concentration in the lesion and contralesion voxels. **(A,B)** Show serial ipsilesional and contralesional NAA concentrations in individual participants, respectively. **(C)** Shows overall trend of significant hemispheric decrease of NAA concentration between 1 and 3 months. Temporally, ipsilesional NAA increased between 3 and 6 months. **(D,E)** Show serial ipsilesional and contralesional choline concentrations in individual participants, respectively. **(F)** Shows an overall significant continual decline of ipsilesional choline concentration between 1 and 6 months, whereas in the contralesion it decreased between 1 and 3 months and then stabilized between 3 and 6 months. **(G,H)** Show serial ipsilesional and contralesional creatine concentrations in individual participants, respectively. **(I)** Illustrates the overall hemispheric and temporal decrease of creatine concentration between 1 and 3 months that stabilized between 3 and 6 months. A correlation between ipsilesional NAA concentration and NIHSS at 1, 3, and 6 months is shown in **(J)**. A significant (*p* < 0.03) correlation was observed at 3 months.

Metabolite changes in individual participants and its average values over the study cohort are summarized in Figure 2. All three metabolites showed statistically significant or clear trends toward lower ipsilesional concentrations compared to the contralesional side at all time points. Statistically significant reductions were found in ipsilesional NAA at 1M and 3M, Cho at 6M, and Cr at 1M and 6M (*p* < 0.03), compared to the contralesional side.

The temporal NAA concentration within the lesion showed an initial decreasing trend between 1 and 3 months (2.88 ± 2.1 to 2.37 ± 1.9 mM, *p* = 0.24) followed by an increase between 3 and 6 (2.37 ± 1.9 to 3.72 ± 2.6 mM, *p* < 0.01) months. The contralesional NAA also showed a strong trend toward lower concentration between 1 and 3 months (5.95 ± 1.0 to 5.09 ± 0.93 mM, *p* < 0.053), but stabilized between 3 and 6 months (5.09 ± 0.93 to 5.17 ± 0.70 mM, *p* = NS). Post graft Cho concentration in the lesion showed a continuing trend for reduction, which reached statistical significance at 6 months (1.62 ± 0.51 to 1.09 ± 0.78 mM, *p* < 0.01), while contralesional measurements revealed a decreasing trend between 1 and 3 months (1.77 ± 0.31 to 1.58 ± 0.49 mM, *p* = NS) that stabilized through 6 months (1.58 ± 0.44 mM). Ipsilesional Cr was stable over the study period, but contralesional Cr reduced between 1 and 3 month (6.23 ± 1.12 to 4.99 ± 0.89 mM, *p* < 0.02).

Ipsilesional NAA concentration and NIHSS score were significantly correlated (*p* < 0.05) at 3 month follow-up and almost reached statistical significance at 6 months (*p* = 0.07), but no correlation was found at 1 month as shown in Figure 2J. There was no statistically significant correlation between Cho, Cr, and NIHSS in either ipsi or contralesional hemisphere. There was no significant correlation between lesion volume change and metabolite concentrations.

## Discussion

A handful of clinical trials have shown safety and feasibility of cell administration as an exploratory new therapeutic option in patients with recent ischemic stroke; however, to the best of our knowledge, this is the first *prospective* study in which patient's cerebral metabolites were serially measured over 6 months after the cellular intervention.

Our finding is in-line with an animal study reporting a significant increase in NAA/Cr and NAA/Cho ratios within the lesion in treated animals with gadolinium labeled mesenchymal stem cells compared to the sham group (39). In another animal study, Qian reported an initial decrease followed by an increase in ipsilesional NAA concentrations without any treatment after inducing transient ischemic stroke (TIA). However, unlike the permanent stroke model, both the lesions and symptoms resolve within a few hours of onset in most patients with TIA (40).

The correlation between the NAA and NIHSS is in support of the previous study (19) suggesting NAA as a marker of therapeutic efficacy. However, the small sample size is the major limitation of our study. Interestingly the decreasing trends of all three metabolites concentrations in the contralesional measurement suggest global depletion of available energy which is supported by a decrease in Cr concentration which is considered a marker of neuronal energy.

Prior studies investigated brain metabolites as a marker of post-stroke recovery especially NAA because it is exclusively found within neurons. A reduction in NAA in the ipsilesional hemisphere has been associated with continued neuronal death and expansion of the infarct (41–43). A continued decline in NAA concentration within the lesion or other ipsilesional tissues at 1, 3, and 6 months has been documented, with few exceptions (44–46). Contrary to the literature, our preliminary results show a trend of regaining NAA concentrations within the lesion between 3 and 6 months following MNC infusion. This is in agreement with a previous study in patients with Parkinson disease who were also treated with MNCs, which reported an increase in NAA in the basal ganglia. Here we found increased NAA within the lesion.

We also noted that most of the patients had one or two broad peaks between 0.9 and 1.5 ppm, resonance regions of lipid and lactate, in the ipsilesional voxel at 1 month (Figure 1) suggesting possible hypoxic environment or cells proliferation which resolved at later follow-up visits. Interestingly, there was no peak between 0.9 and 1.5 ppm in the contralesional voxel. Further investigation of this finding requires a non-treated matching control patients as a control group.

A higher choline concentration around the penumbra region in acute phase has been documented (47). Here we observed a decreasing trend of choline concentration in both ipsilesional and contralesional measurement between 1 and 3 months. While the choline signal stabilized in the contralesional side, it continued to decrease in the lesion suggestion progressive membrane disintegration. The opposite trend between NAA (increasing) and Cho (decreasing) between 3 and 6 months in the lesion could be due to a higher number of immature neurons without membrane, which is typically developed at the final stages of neuronal maturation. However, we will investigate this hypothesis in future animal study combining MRS and histology.

## Study Limitation

The limitations of this exploratory pilot study included the small sample size, the absence of non-treated patient group, the limited coverage of single voxel spectroscopy, and known partial volume effects. Future studies will address these limitations by increasing sample size, adding non-treated patients group and obtaining multi-voxel MRS.

## Conclusion

Our study supports using MRS as an innovative means to evaluate treatment efficacies by quantifying lesion metabolites. In this preliminary study we observed signals of a slow treatment effect, regain in NAA concentrations in a damaged tissue area, and patients with increased NAA recovering better on clinical deficits scores.

## Author Contributions

MH designed the study, carried MRI quantitative analysis, and drafted the manuscript. RG contributed to data analysis, assisted in optimizing MRI acquisition. SG and SB created plots and figures. SA and FV recruited, consented, and obtained patient's serial neurological assessment. XZ performed statistical analysis. KH and PN provided qualitative and quantitative quality assurance of images and data analysis. OA, EF, and CS provided radiology reports and assisted in lesion locations. SS supervised the study and provided necessary resources. All authors contributed to and approved the final manuscript.

### Conflict of Interest Statement

The authors declare that the research was conducted in the absence of any commercial or financial relationships that could be construed as a potential conflict of interest.
